# Genome-wide association study of subcortical brain volume in PTSD cases and trauma-exposed controls

**DOI:** 10.1038/s41398-017-0021-6

**Published:** 2017-11-30

**Authors:** Rajendra A. Morey, Sarah L. Davis, Melanie E. Garrett, Courtney C. Haswell, Mid-Atlantic MIRECC Workgroup, Christine E. Marx, Jean C. Beckham, Gregory McCarthy, Michael A. Hauser, Allison E. Ashley-Koch

**Affiliations:** 1 Mid-Atlantic Mental Illness Research Education and Clinical Center, Durham VAMC, Durham, NC USA; 2 Department of Psychiatry and Behavioral Sciences, Duke University, Durham, NC USA; 3 Duke-UNC Brain Imaging and Analysis Center, Duke University, Durham, NC USA; 4 Department of Psychology, Yale University, New Haven, CT USA; 5 Duke Molecular Physiology Institute, Durham, NC USA

## Abstract

Depending on the traumatic event, a significant fraction of trauma survivors subsequently develop PTSD. The additional variability in PTSD risk is expected to arise from genetic susceptibility. Unfortunately, several genome-wide association studies (GWAS) have failed to identify a consistent genetic marker for PTSD. The heritability of intermediate phenotypes such as regional brain volumes is often 80% or higher. We conducted a GWAS of subcortical brain volumes in a sample of recent military veteran trauma survivors (*n* = 157), grouped into PTSD (*n* = 66) and non-PTSD controls (*n* = 91). Covariates included PTSD diagnosis, sex, intracranial volume, ancestry, childhood trauma, SNP×PTSD diagnosis, and SNP×childhood trauma. We identified several genetic markers in high linkage disequilibrium (LD) with rs9373240 (*p* = 2.0 × 10^−7^, FDR *q* = 0.0375) that were associated with caudate volume. We also observed a significant interaction between rs9373240 and childhood trauma (*p*-values = 0.0007–0.002), whereby increased trauma exposure produced a stronger association between SNPs and increased caudate volume. We identified several SNPs in high LD with rs34043524, which is downstream of the *TRAM1L1* gene that were associated with right lateral ventricular volume (*p* = 1.73 × 10^−7^; FDR *q* = 0.032) and were also associated with lifetime alcohol abuse or dependence (*p* = 2.49 × 10^−7^; FDR *q* = 0.0375). Finally, we identified several SNPs in high LD with rs13140180 (*p* = 2.58 × 10^−7^; FDR *q* = .0016), an intergenic region on chromosome 4, and several SNPs in the *TMPRSS15* associated with right nucleus accumbens volume (*p* = 2.58 × 10^−7^; FDR *q* = 0.017). Both *TRAM1L1* and *TMPRSS15* have been previously implicated in neuronal function. Key results survived genome-wide multiple-testing correction in our sample. Leveraging neuroimaging phenotypes may offer a shortcut, relative to clinical phenotypes, in mapping the genetic architecture and neurobiological pathways of PTSD.

## Introduction

Most individuals in the general population are exposed to at least one traumatic event in their lifetime^1^, but only a fraction of trauma survivors subsequently develop posttraumatic stress disorder (PTSD)^2^. Thus, exposure to traumatic events is a necessary, but insufficient environmental risk factor for developing PTSD. The developmental timing of the traumatic events appears to be important since exposure to trauma or adversity in childhood increases the risk of developing PTSD following trauma in adulthood^3^. Family and twin heritability studies have demonstrated that genetic risk also contributes to the development of PTSD. In order to identify putative PTSD genetic loci, several groups have conducted unbiased genome-wide association studies (GWAS) based on the clinical diagnosis of PTSD^4–8^. These GWAS have had limited success. Among the loci that were implicated, the effect sizes on PTSD risk were relatively large for a complex disease, with odds ratios ranging from 1.4 to 3.7. However, despite these putatively large genetic effects, each GWAS identified different loci. None of the top hits (*p < *10^−5^) for the studies overlapped except for *RORA*, which demonstrated a modest degree of replication^9^. The lack of replication in PTSD GWAS could be explained by differences among the study participants with respect to trauma type, trauma severity, ethnicity, and/or gender, among other distinctions. Additionally, it is expected that PTSD has a complex genetic architecture with polygenic and gene×environment influences. These underlying complexities likely have also contributed to the heterogeneity of the GWAS findings. A major component of the genetic heterogeneity is the clinical heterogeneity that is inherent in psychiatric nosology and the subjective approach to assessment of PTSD symptoms (patient-reported behaviors).

One approach to mitigating the clinical heterogeneity in PTSD and other neuropsychiatric disorders is to utilize quantitative brain-based neuroimaging phenotypes. A variety of brain measures from fMRI, DTI, and functional connectivity have been associated with candidate genes in PTSD^10–13^, but use of GWAS with brain measures in PTSD remains largely unexplored. A large neuroimaging GWAS of healthy (non-clinical) individuals (*n* = 30,717) revealed four novel genetic variants that modulated subcortical brain volume in the putamen: (1) an intergenic locus 50 kilobases (kb) downstream of the *KTN1* gene (rs945270) that encodes the protein kinectin involved in organelle transport; (2) an intronic locus within *DCC* (rs62097986) that encodes a netrin receptor involved in axon guidance and migration; (3) an intronic locus within *BCL2L1* (rs6087771) that inhibits programmed cell death in immature neurons; and, (4) an intronic locus within *DLG2* (rs683250) that encodes a protein that organizes postsynaptic density. Caudate volume was associated with an intergenic locus 80 kb from *FAT3* (rs1318862) that influences neuronal morphology during embryonic development. Hippocampal volume was associated with two loci: (1) an intergenic locus near the *HRK* gene (rs77956314), and (2) an intronic locus in the *MSRB3* gene (rs61921502)^14,15^. Brain connectivity assessed at the connectome-wide level with GWAS in healthy individuals also identified an association with *SPON1* (rs2618516) that contributes to growth and guidance of axons in the spinal cord^16^.

Genetic variation is expected to exert an enduring effect on brain structures, which are ultimately associated with behavior and predisposition to disease^14^. In this way, genetic risk factors for psychiatric diagnoses such as PTSD may be more easily detected by examining intermediate phenotypes such as brain measures obtained from MRI^17^. Intermediate phenotypes, or endophenotypes, may have a simpler genetic architecture than the PTSD clinical diagnosis^18^. Brain measures also offer a more precise, objective, and reproducible phenotype than a clinical diagnostic scale^19^. Moreover, the quantitative aspect of most endophenotypes such as brain morphometry or volumetry provide a boost in statistical power compared with qualitative traits such as PTSD diagnosis. This is true for targeted candidate gene analyses, as well as for GWAS.

We approached identifying a suitable endophenotype for PTSD by turning to the most consistently replicated neuroimaging finding, which is the association of PTSD with a smaller hippocampus^20–22^. The amygdala, perhaps the most strongly implicated structure in PTSD based in part on hypothesized mechanisms for PTSD, showed reduced volume in PTSD patients from a meta-analysis^20^ as well as a subsequent study we conducted in a large combat–veteran cohort^23^. The anterior cingulate, a medial prefrontal cortical structure, shows compromised voxel-based morphometry associated with PTSD^24–26^. These, and other successes associating genetic markers with variability in brain measures for other psychiatric disorders^27–29^, as well as parallel efforts establishing differences in subcortical brain volumes in PTSD, spurred our investigation of subcortical brain volumes on a genome-wide level in PTSD, which remain conspicuously unexplored. We hypothesized a modulation of subcortical brain volume, particularly hippocampus and amygdala, by genetic markers in PTSD patients and trauma-exposed controls^23^. We pursued a GWAS approach despite our small sample size because of the concerns with candidate gene approaches that can produce high rates of spurious associations^30,31^.

## Methods

### Participants and clinical measures

Participants (*n* = 157) from a repository (Mid-Atlantic MIRECC, Durham NC) of Iraq and Afghanistan era military service members who contributed blood for genotyping, clinical assessment data, and MRI scans were analyzed. Participants were screened for inclusion/exclusion criteria based on information available in the repository. Important exclusions included psychotic symptoms, high risk of suicide, contraindication to MRI, current substance abuse, neurological disorders, and age over 65 years. To reduce the effects of population stratification in a multi-racial sample, analyses were limited to non-Hispanic black (NHB; *n* = 74) and non-Hispanic white (NHW; *n* = 83) participants from these studies who consented to the genetic and imaging components and had data available at the time of analysis. All participants provided written informed consent to participate in procedures reviewed and approved by the Institutional Review Boards at Duke University and the Durham VA Medical Center. Participants completed questionnaires assessing traumatic life events (Traumatic Life Events Questionnaire, TLEQ^32^), combat exposure (Combat Exposure Scale, CES^33^), and depressive symptoms (Beck Depression Inventory-II, BDI-II^34^). PTSD diagnosis was ascertained with the Structured Clinical Interview for DSM-IV (SCID) and confirmed with the Clinician-Administered PTSD Scale^35^(CAPS) in 152 (97%) participants and with the Davidson Trauma Scale^36^(DTS) in 5 (3%) participants. Alcohol abuse and dependence was determined by the SCID.

### MRI acquisition

Images were acquired on a General Electric 3-Tesla Signa EXCITE scanner equipped with an 8-channel headcoil. High-resolution T1-weighted whole-brain images with 1-mm isotropic voxels using array spatial sensitivity encoding technique (ASSET) and fast spoiled gradient-recall (3D-FSPGR) were acquired axially for all participants. Image parameters were optimized for contrast between white matter, gray matter, and CSF (TR/TE/flip angle = 7.484 ms/2.984 ms/12°, FOV = 256 mm, 1-mm slice thickness, 166 slices, 256 × 256 matrix, 1 excitation).

### Image analysis

All T1 images were visually inspected (CCH, SLD) to assure appropriate quality. Automated segmentation and labeling of the left and right subcortical structures and estimation of total intracranial volume from participants’ T1 images were performed using the FreeSurfer image analysis suite^37^ (version 5.3.0; http://surfer.nmr.mgh.harvard.edu/) and its library tool *recon-all* (Supplementary Materials). We applied standardized protocols for image analysis and quality assurance developed by ENIGMA, which are openly available online (http://enigma.ini.usc.edu/protocols/imaging-protocols/). All participants passed this inspection process without the need for manual adjustment.

Subcortical volumes for the left and right hemispheres were generated in each subject for the lateral ventricle, thalamus, caudate, putamen, pallidum, hippocampus, amygdala, and accumbens. Spatial normalization by affine registration to Talairach space and skull stripping were performed on the T1 images. Registration was checked visually for accuracy (CCH, SLD). FreeSurfer segmentation and labeling of subcortical structures was based on a combination of voxel intensity, probabilistic atlas location, and the spatial relationships of the voxels to the location of nearby subcortical structures. Using the FreeSurfer library function *mri_label2vol* and a transformation matrix generated by *tkregister2*, the segmentation labels were returned to native space. The native-space segmentations were converted to LAS orientation and then the subcortical structures were extracted using the segmentation labels.

### Genotyping

Samples (*n* = 157) obtained from a larger parent study of 2312 PTSD cases and controls were genotyped as previously described^38^. Briefly, DNA was extracted from whole blood and was genotyped in batches using three different Illumina BeadChips (Illumina, San Diego, CA). Quality control was assessed separately in each batch and samples with low call rates were excluded from further analysis. A global reference panel from 1000 Genomes was used to impute missing genotypes in each batch separately; imputed SNPs with certainty <0.90 were excluded. Overlapping SNPs in the imputed NHB and NHW subsets were merged to create a final data set comprising 2,711,511 SNPs.

### Statistical analysis

Principal components (PC) analysis was run using the *smartpca* program from the software package EIGENSOFT^39^ to assess population stratification. Only one PC was necessary to account for the variability observed in this subset of individuals, essentially identifying the NHB and the NHW subjects. Linear regression was utilized using PLINK^40^ to test for association between subcortical volume and each SNP, assuming an additive genetic model. Left and right brain hemisphere volumes were analyzed separately. All subcortical volumes were normally distributed except the lateral ventricles, which were log-transformed for analysis. Covariates included sex, age, one PC, lifetime PTSD diagnosis, intracranial volume, and childhood trauma (0 = no childhood trauma; 1 = exposure to a single category of childhood trauma; 2 = exposure to two or more categories of childhood trauma, as reported from TLEQ items 12, 13, 15, 16, and 17). To reduce redundancy in this imputed data set, we used the linkage disequilibrium (LD) clumping method in PLINK, choosing an r2 threshold of 0.25 and a 500 kb window, as reported previously^41^. False discovery rate (FDR) *q*-values were generated using PROC MULTTEST in SAS version 9.4 (SAS Systems, Cary, NC). The FDR correction was applied only across SNPs, but not across the eight subcortical regions and two hemispheres because of the inherent correlation between regional volumes. The known associations between these measures would produce a higher rate of significant associations than expected, and therefore an FDR correction would lead to overly conservative inferences. In addition, a multiple-comparison correction for brain structures previously implicated in PTSD that include amygdala^20,23,42^, caudate^43–45^ hippocampus^20,23^, lateral ventricles^46–48^, thalamus^49,50^, and nucleus accumbens^51,52^ would also contribute to overly conservative inferences. Post hoc interaction analyses were performed for the SNPs with significant main effects: SNP×childhood trauma and SNP×lifetime PTSD interactions were investigated for association with brain volume. Plots of significant SNP associations with LD structure by region were generated using LocusZoom^53^. Manhattan plots and Q–Q plots were produced using the R package *qqman* ^54^ (Supplementary Figs. 1 and 2).

We conducted gene ontology analysis to obtain a more comprehensive picture of the biological pathways that may be implicated in modulating subcortical volume following trauma exposure. We used the Genomic Regions Enrichment of Annotations Tool (GREAT), given that many of the SNPs associated with brain volume in our data set were intergenic^55^. The analysis utilized nominally significant SNPs (*p* < 0.001) for the four brain regions showing FDR-significant results (right lateral ventricle, right caudate, right pallidum, and right accumbens).

## Results

Important clinical and sociodemographic information is reported by diagnostic (PTSD and non-PTSD) and ancestry (NHB and NHW) groups in Table 1. The PTSD group did not differ significantly from the control group with respect to age, gender, ethnicity, childhood trauma exposure, substance use disorders, or alcohol use disorders. The NHB group did not differ from the NHW group with respect to any of these parameters except that a significantly larger proportion of the NHB group was female compared to the NHW group (24.3% vs. 8.4%, *p* = 0.01).

**Table 1:** Demographic and clinical information by diagnostic and ancestry groups

Characteristic	PTSD (*n* = 66)	Non-PTSD (*n* = 91)	Group comparison	NHW (*n* = 83)	NHB (*n* = 74)	Group comparison
Age, mean (SD)	39 (9.65)	40.01 (10.32)	*p* = 0.51	38.40 (10.24)	41.0 (9.66)	*p* = 0.11
Gender, no. of females (%)	13 (19.7)	12 (13.19)	*p* = 0.27	7 (8.4)	18 (24.3)	*p* = 0.01
Race, no. of Caucasian (%)	30 (45.45)	53 (58.24)	*p* = 0.12	83(100)	0	NA
Child trauma category 0, 1, ≥2 (%)	27 (41.0), 22 (33.3), 17 (25.8)	45 (49.5), 26 (28.6), 20 (22.0)	*p* = 0.57	43 (51.8), 22 (26.5), 18 (21.7)	29 (39.2), 26 (35.1), 19 (25.7)	*p* = 0.28
Alcohol abuse/dependency (%)	21 (32.8)	28 (30.77)	*p* = 0.79	25 (30.12)	24 (33.33)	*p* = 0.67
SCID-IV lifetime PTSD diagnosis (%)	66 (100)	0	NA	30 (36.14)	36 (48.65)	*p* = 0.11

*SD* standard deviation, *no*. number, *AUDIT* Alcohol Use Disorders Identification Test, *SCID-IV* Structured Clinical Interview for DSM-IV, *CAPS-IV* Clinician Administered PTSD Scaler with DSM-IV criteria

### Association with caudate volume

**Fig. 1:**
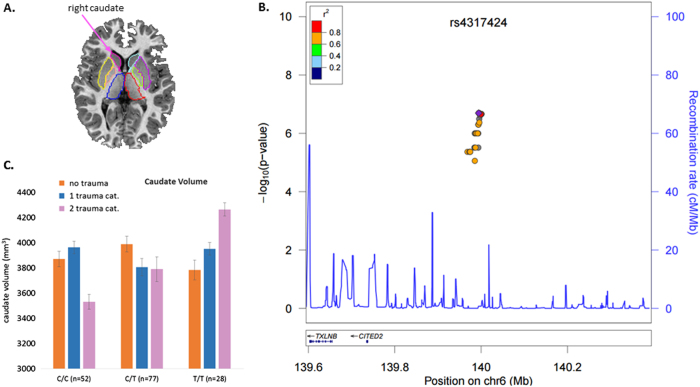
Several genetic markers in high LD with rs4317424 associated with right caudate volume **a** Caudate volume segmented from structural MRI by FreeSurfer. **b** Locus zoom plot for SNPs in the region surrounding rs4317424. All SNPs are plotted with their discovery sample *p*-values against genomic position according to their pairwise correlation (*r* ^2^) with the signal SNP. The blue line represents the estimated recombination rate. Gene annotations are shown as dark lines along the *x* axis. **c** The association of SNP rs9373240 on chromosome 6 with R-caudate volume showed a significant interaction with childhood trauma exposure (*p* = 0.0007 with increasing categories of childhood trauma exposure. The Q–Q plots and Manhattan Plots are in Supplementary Figs. 1 and 2

The R-caudate (Fig. 1a; Supplementary Figs. 1a and 2a) volume was significantly associated with a region on chromosome 6 comprising many intergenic SNPs in high LD, rs4317424 was the most strongly associated SNP with a beta value of 236.6; for each additional C allele, mean R-caudate volume increased by 236.3 mm^3^ (*p* = 2.0 × 10^−7^, FDR *q* = 0.0375 (Table 2). Figure 1b displays the strength and extent of these association signals relative to genomic position, local LD, and the positions of genes in the region. Two correlated, intronic SNPs in the *NKAIN3* gene on chromosome 8 were also associated with R-caudate volume such that for each additional minor allele, mean R-caudate volume increased (rs34720850, beta = 423.5 mm^3^, *p* = 8.3 × 10^−7^, FDR *q* = 0.077). The SNPs on chromosome 6 also interacted with childhood trauma exposure to influence R-caudate (*p*-values = 0.0007–0.002). Further exploration of this interaction revealed that the associations of these SNPs with R-caudate volume became stronger with increasing exposure to childhood trauma (Fig. 1c). For example, rs9373240 significantly interacts with childhood trauma exposure to predict R-caudate volume (*p* = 0.0007). Among subjects with no childhood trauma exposure, rs9373240 is not associated with R-caudate volume (*p* = 0.132). Among subjects who endorsed one category of childhood trauma exposure, rs9373240 is associated with R-caudate volume (*p* = 0.007); however, among those subjects who endorsed two or more categories of childhood trauma, the association is much more robust (*p* = 6.36 × 10^−8^). None of the SNPs on chromosome 6 interacted significantly with PTSD diagnosis, and neither of the SNPs in *NKAIN3* showed a significant interaction with childhood trauma or PTSD diagnosis.

**Table 2:** Top SNP associations with subcortical brain volumes, regressors, and interactions

		Main effects			Interactions	
Subcortical structure	SNP	SNP *p*-value	FDR *p*-value	Alcohol D/O *p*-value	PTSD×SNP *p*-value	Childhood trauma×SNP *p*-value
R-lateral ventricle	rs34043524	1.73 × 10^−7^	0.032	2.49 × 10^−7^	0.298	0.080
R-caudate	rs4317424	2.02 × 10^−7^	0.0375		0.455	0.00156
R-accumbens	rs55886168	6.08 × 10^−8^	0.011		0.510	0.869
R-pallidum	rs55685119	7.36 × 10^−8^	0.014		0.527	0.157

*DO* disorder, *SNP* single-nucleotide polymorphism, *R* right, *PTSD* posttraumatic stress disorder

### Association with lateral ventricle volume

**Fig. 2:**
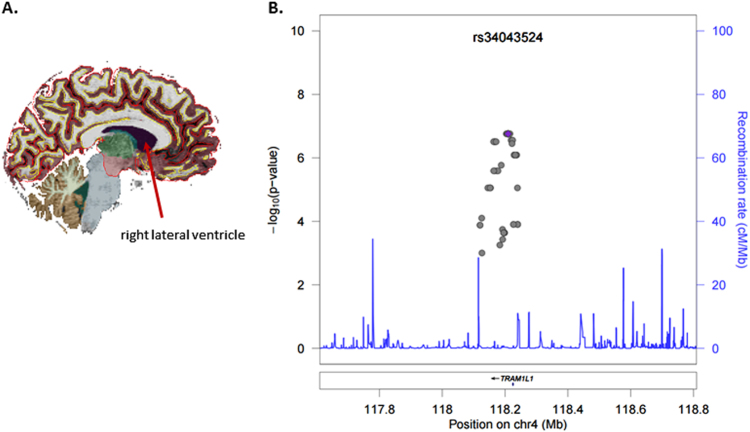
Several genetic markers in high LD with rs34043524 associated with lateral ventricle volume **a** Lateral ventricle cartoon (replace with MRI). **b** Locus zoom plot for SNPs in the region surrounding rs34043524. All SNPs are plotted with their discovery sample *P*-values against genomic position according to their pairwise correlation (*r* ^2^) with the signal SNP. The blue line represents the estimated recombination rate. Gene annotations are shown as dark lines along the *x* axis. The Q–Q plots and Manhattan Plots are in Supplementary Figs. 1 and 2

The R-lateral ventricle (Fig. 2a; Supplementary Figs. 1b and 2b) volume was significantly associated with a region on chromosome 4 comprising many correlated SNPs, downstream of *TRAM1L1*. The most significant SNP associated with R-lateral ventricle was rs34043524, such that for each additional G allele, R-lateral ventricle volume decreased. Figure 2b displays the association of SNPs near *TRAM1L1* with R-lateral ventricle. The *TRAM1L1* locus in the 4q21-q32 region has been previously hypothesized to play a role in alcohol dependence, but prior investigation produced negative results^56^. This prompted us to conduct follow-up analysis for association with alcohol dependence (i) as an additional covariate in the association between genotype and caudate volume, and (ii) as a phenotype (main effect) associated with genotype^56^. We did, in fact, observe that the same SNPs downstream of *TRAM1L1* that were associated with R-lateral ventricle volume were also associated with lifetime alcohol abuse or dependence in our data set (*p*-values = 1.92 × 10^−7^ to 2.84 × 10^−6^). Importantly, inclusion of lifetime alcohol abuse or dependence as a covariate in the model of SNPs predicting R-lateral ventricle volume did not attenuate the association. None of the SNPs downstream of *TRAM1L1* significantly interacted with either PTSD diagnosis or childhood trauma exposure to predict R-lateral ventricle volume.

### Association with nucleus accumbens volume

**Fig. 3:**
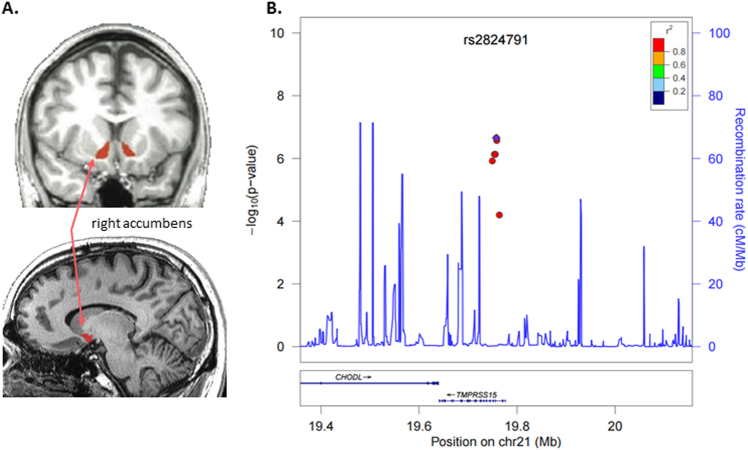
Several genetic markers in high LD with rs2824791 associated with R-nucleus accumbens volume **a** R-nucleus accumbens volume segmented from structural MRI by FreeSurfer. **b** Locus zoom plot for SNPs in the region surrounding rs2824791. All SNPs are plotted with their discovery sample *p*-values against genomic position according to their pairwise correlation (*r* ^2^) with the signal SNP. The blue line represents the estimated recombination rate. Gene annotations are shown as dark lines along the *x* axis. The Q–Q plots and Manhattan Plots are in Supplementary Figs. 1 and 2

The most significant SNP associated with R-accumbens (Fig. 3a; Supplementary Figs. 1c and 2c **)** volume was rs55886168, which is located on chromosome 20 in a long non-coding RNA (LINC01522). For each additional T allele, R-accumbens volume increased by 79.33 mm^3^ (*p* = 6.1 × 10^−8^, FDR *q* = 0.011). There were also several correlated, intronic SNPs in *TMPRSS15* on chromosome 21 associated with R-accumbens volume (*p* = 2.58 × 10^−7^; FDR *q* = 0.016). A plot of these SNPs is shown in Fig. 3b. Other SNPs associated with R-accumbens volume included intergenic SNPs in high LD with rs13140180 on chromosome 4 (*p* = 2.58 × 10^−7^; FDR *q* = 0.016), and high LD with rs4053747 chromosome 6 (*p* = 3.80 × 10^-7^; FDR *q* = 0.018). None of the SNPs associated with R-accumbens volume displayed significant interactions with either PTSD diagnosis or childhood trauma exposure.

We performed gene ontology analysis with GREAT using nominally significant SNPs (*p* < 0.001) for the four brain regions showing FDR significance (right lateral ventricle, right caudate, right pallidum, and right accumbens). We identified several significant gene ontology terms for SNPs associated with right lateral ventricle, right caudate, and right pallidum that are detailed in Supplementary Tables 1, 2, and 3, respectively.

## Discussion

We performed high-density genome-wide association analysis of subcortical brain structures segmented by FreeSurfer in trauma-exposed US military veterans with and without a PTSD diagnosis. We identified several SNPs (in high LD with rs9373240) in an intergenic region on chromosome 6 that were associated with caudate volume. These SNPs also significantly interacted with childhood trauma, whereby increasing trauma exposure produced a stronger association between SNPs and caudate volume. While these SNPs do not reside within a gene, it is possible that they function as regulatory markers. Caudate volume was also modulated by SNPs in *NKAIN3* (rs34720850), which codes for a Na+/K+ transporting ATPase interacting 3 protein. The protein is a member of the NKAIN family of mammalian proteins that are neuronally expressed in multiple regions of the mouse brain. In particular, NKAIN3 is strongly expressed in the hippocampus^57^. Previously, *NKAIN3* has been reported in bulimia nervosa and taste perception of salt^58,59^. Structural imaging volumetry of the caudate in PTSD has been limited to studies with small sample sizes, among other limitations, making it challenging to draw strong conclusions from the existing literature. Smaller caudate volume has been associated with early life stress^45^, with higher CAPS scores among combat-exposed veterans^43^. Conversely, larger caudate volumes have been associated with PTSD^44^, while many other studies including the present sample showed no such association^60,61^. Abnormal functioning of the caudate has been established in PTSD with generally lower caudate (dorsal striatum) activation to rewards and lower deactivation to losses (punishment) as compared to trauma controls^51,62–64^. The findings are consistent with hyporesponsivity to positive stimuli and anhedonia, which are common in PTSD^65^. Relatedly, abnormalities in D2 dopamine receptors located in the striatum have been observed in PTSD^66^. Diminished hedonic tone and reward responsivity are prominent features of depression, which has a more firmly established and extensively investigated association with caudate dysfunction^67^. However, depressive symptoms are highly comorbid in most PTSD samples and mounting empirical evidence questions a distinct nosology between the two disorders^68^.

Additionally, we found that nucleus accumbens volume was modulated by several SNPs in high LD with rs13140180 on chromosome 4, rs1577238 on chromosome 6, and *TMPRSS15*. The accumbens (ventral striatum) plays a major role in reward and reinforcement learning and a secondary role in processing fear, which are central to the prevailing behavioral models of PTSD^51,69^. The *TMPRSS15* gene codes for a transmembrane protease serine 15, an enzyme that converts proenzyme trysinogen to trypsin. Trypsin-like serine proteases and trypsin play very important roles in neural development, neuroregeneration, and synaptic plasticity, particularly memory formation^70,71^. Accumulating evidence suggests that the brain has co-opted the activities of enteropeptidases, which are central to digestive function, to regulate various processes underlying synaptic activity and behavior. Enteropeptidases protect hippocampal neurons from death induced by glutamate toxicity^72^.

Finally, we identified several SNPs (in high LD with rs34043524) downstream of the *TRAM1L1* gene on chromosome 4 that were associated with right lateral ventricular volume. Previously, an association of *TRAM1L1* and alcohol dependence was hypothesized by Kalsi et al.^56^ based on LOD score from genetic linkage analysis. We identified a significant association (*p* = 1.9 × 10^−7^) between rs34043524 and clinical diagnosis of alcohol use disorder. Importantly, the association between the same SNP and ventricular volume remained significant after adding alcohol dependence as a covariate (*p* = 2.5 × 10^−7^), which suggests the association of this SNP with lateral ventricular volume and with alcohol dependence are independent of each other. Lateral ventricle volumetry lacks consistent results in PTSD. Early-life maltreatment that subsequently develops into PTSD was associated with larger lateral ventricular volume^46,73^. The presence of lateral ventricular volume differences reported in children with PTSD, but the lack thereof in adults deserves mention. Bipolar disorder^74^ and schizophrenia^75^ are consistently associated with enlarged lateral ventricle volume. The SNP associated with the lateral ventricle volume significantly interacts with PTSD diagnosis and was not identified in an extremely large normative sample (n~31,000) of subcortical volumes, suggesting the SNP could be a non-specific genetic marker for psychopathology.

The three genes *NKAIN3*, *TRAM1L1*, and *TMPRSS15* have been previously implicated in neuronal function^56,70,71^, increasing the potential biological relevance of our findings. Indeed, the latest evidence implicates a common genetic architecture spans common neuropsychiatric disorders. A large GWAS (*n* ~ 60,000) by the Psychiatric Genetics Consortium (PGC) found common neuronal, immune, and histone pathways across major depression, bipolar disorder, and schizophrenia. Risk variants for psychiatric disorders aggregate in particular biological pathways and these pathways are often shared between disorders suggesting a shared etiology among disorders and the co-heritability of multiple psychiatric disorders^76^. Calculations using common SNPs found high correlations between schizophrenia and bipolar disorder (*h* ^2^ = 0.68), major depressive disorder and bipolar disorder (*h* ^*2*^ = 0.47), as well as schizophrenia and major depressive disorder (*h* ^2^ = 0.43)^76^. It is certainly possible that the current classification of psychiatric disorders as clinical syndromes with a constellation of behavioral symptoms may serve to inflate the apparent pleiotropy compared to an alternate nosology constructed on pathomechanistic markers of etiology^77^. Thus, genome wide associations of brain measures hold the promise of moving us one step closer to this new psychiatric nosology^17,78^. On the other hand, attempts to find disease-associated genetic variation that point to molecular mechanisms of pathogenesis has been further complicated by the polygenicity of clinical diagnoses and phenotypes^18,79^. Thus, the effects of an overall polygenic risk profile score on neuroimaging abnormalities might help to identify genetic susceptibility or genetically mediated traits of PTSD^80,81^.

### Strengths and limitations

It is important to consider the value of imaging genetics approach and its potential relevance to PTSD research, particularly in the translational and clinical domains. The first goal is simply looking for an association between genetic markers and neuroimaging phenotypes in the context of a PTSD sample. Any difference in the SNP associations with a particular neuroimaging measure could be explained by the presence of PTSD. It is possible that the neuroimaging phenotype is assessing an underlying dimension of PTSD not captured or accessible by its clinical diagnosis and may explain, at least in part, the lack of SNP×PTSD interaction finding in the present sample. However, a limitation of our analysis of interaction effects was that it was restricted to SNPs that demonstrated significant main effects because of concern that our sample was too small to pursue interaction effects at the genome-wide level. Alternatively, a significant interaction can be further explored by testing the association between genetic markers and brain imaging measures that are different in PTSD cases than controls^10^.

Factors contributing to negative results for the amygdala include limited accuracy of FreeSurfer segmentation as compared to hand-tracing whereas the hippocampus and other structures are more accurate^82^. The concern with accuracy introduces added variance that can be overcome by increasing the sample size in equal measure^82^. Factors contributing to negative results for the hippocampus include the heterogeneity of a structure comprising several subfields that are differentially affected by PTSD. Specific subfields, namely cornu ammonis 1 (CA1), cornu ammonis 3 (CA3), and dentate gyrus, show volume reduction in PTSD^83^, but including other subfields may confound associations with overall hippocampal volume, which was utilized in this study. Likewise, the amygdala contains several subregions (nuclei) that are differentially involved in PTSD based on their functional roles. Advanced methods that have recently become available for segmenting the hippocampus^84^ and amygdala^85^ into functional subunits may hold greater promise as phenotypes for identifying genetic markers associated with PTSD.

Another limitation of the present study was the small sample size, rendering the results susceptible to type-1 error. The presence of two ancestry subgroups in our sample, which we controlled statistically with a single principal component, was considered as a minor limitation because separate analyses of these two racial groups yielded results consistent with the original analyses of the combined groups. Thus, we do not anticipate that population stratification is an explanation for the present results.

## Conclusion

Ultimately the promise of finding genetic determinants of PTSD is that they signal the presence of etiologic pathways for which targeted interventions may be devised and deployed. Attempts to find disease-associated genetic variation that point to molecular mechanisms of pathogenesis has proven challenging due to the polygenicity of clinical phenotypes^18,79^. Leveraging neuroimaging phenotypes may offer a shortcut over clinical phenotypes in identifying these elusive genetic markers and relevant neurobiological pathways^86,87^.

## Electronic supplementary material

Supplemental Figure 1

Supplemental Figure 2

Supplemental Tables
