# Supplementary figures and images for: Genome-wide association study of subcortical brain volume in PTSD cases and trauma-exposed controls
Source: Transl Psychiatry. 2017 Nov 30;7:1265. doi: 10.1038/s41398-017-0021-6 (PMC5802459; doi:10.1038/s41398-017-0021-6)

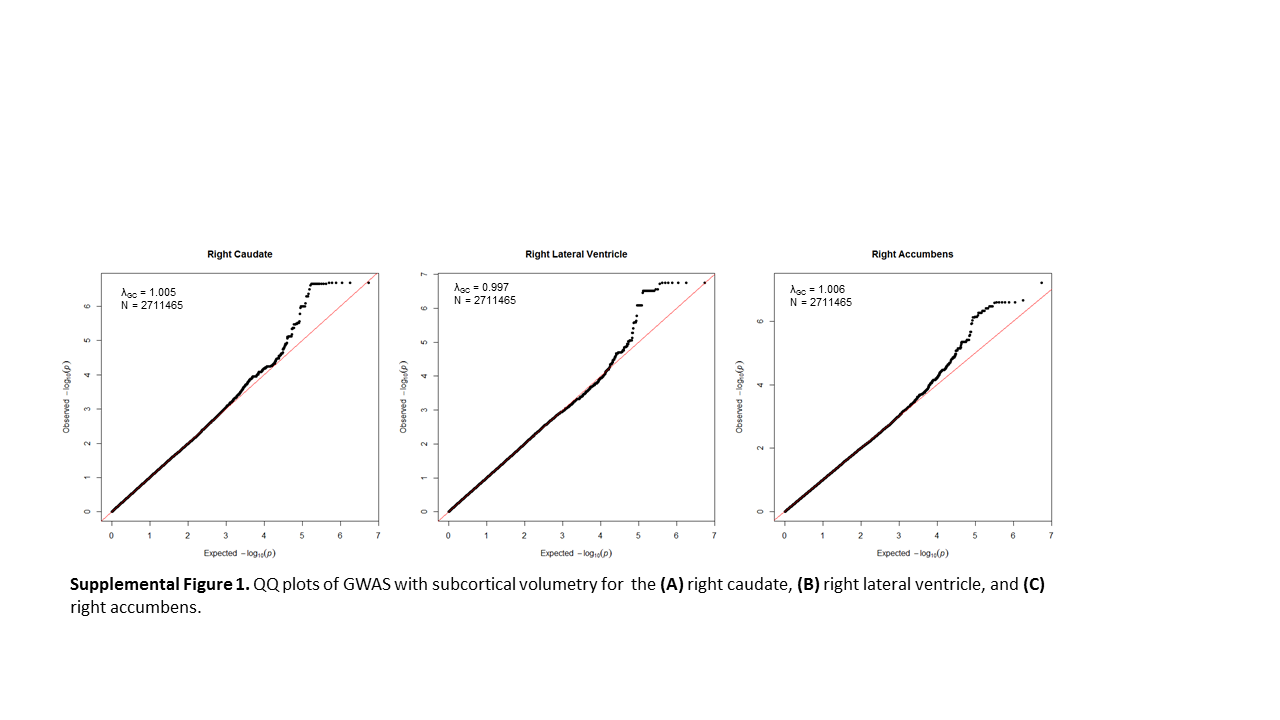

Supplement: Supplementary file 1 — Supplemental Figure 1 [file 41398_2017_21_MOESM1_ESM.tif]

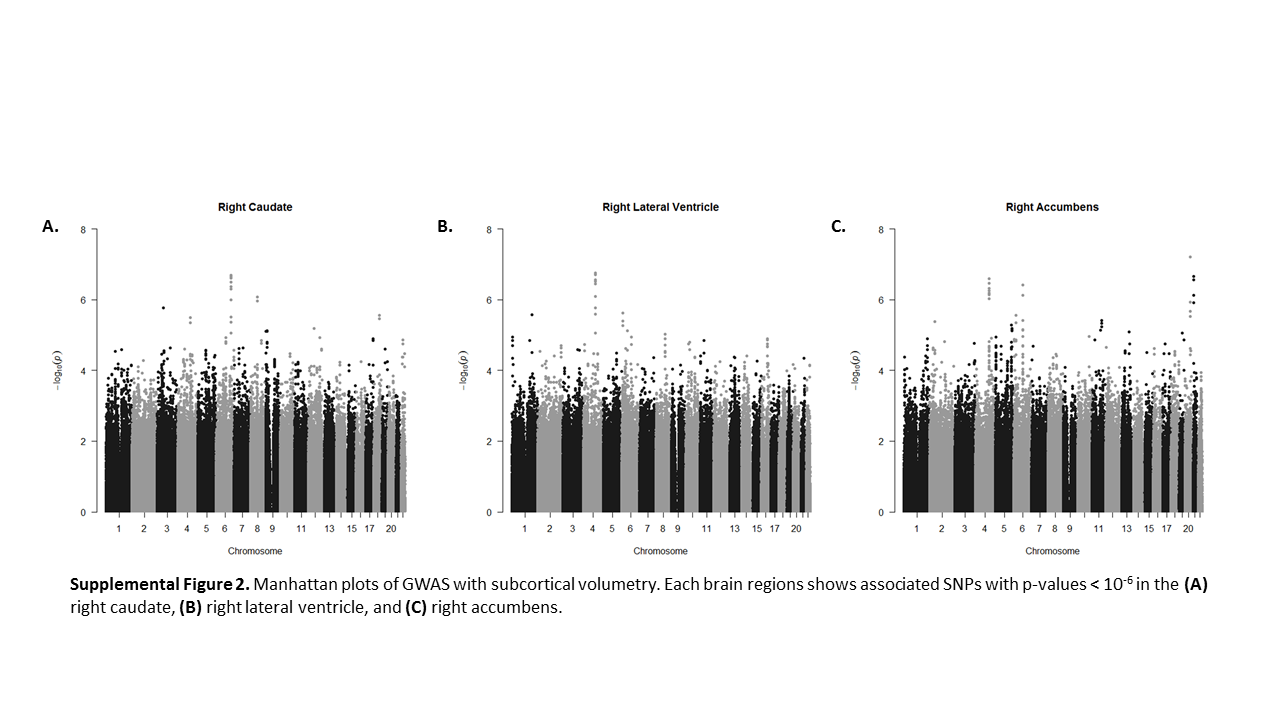

Supplement: Supplementary file 2 — Supplemental Figure 2 [file 41398_2017_21_MOESM2_ESM.tif]
