# Supplementary material for: Genome-wide association study of subcortical brain volume in PTSD cases and trauma-exposed controls
Source: Transl Psychiatry. 2017 Nov 30;7:1265. doi: 10.1038/s41398-017-0021-6 (PMC5802459; doi:10.1038/s41398-017-0021-6)
Supplement: Supplementary file 3 — Supplemental Tables [file 41398_2017_21_MOESM3_ESM.docx]

**Supplemental Table 1.** Results of Gene Ontology for Right Lateral Ventricle

**Supplemental Table 2.** Results of Gene Ontology for Right Caudate Nucleus

**Supplemental Table 3.** Results of Gene Ontology for Right Pallidum
